# Nasopharyngeal Branchial Cyst and Thornwaldt Cyst in a Patient with Hearing Loss after Coronavirus Infection

**DOI:** 10.22038/IJORL.2023.71639.3436

**Published:** 2023-09

**Authors:** Hosna Zobairy, Shahin Rajaeih, Mersad Mehrnahad, Parisa Modaresi, Ahmad Sofi-Mahmudi

**Affiliations:** 1 *Department of Otolaryngology, Kurdistan University of Medical Science, Sanandaj, Iran. *; 2 *ENT and Head and Neck Research Center and Department, the Five Senses Health Institute, Firoozgar Hospital, Iran University of Medical Sciences, Tehran, Iran. *; 3 *Department of Radiology, Arak University of Medical Sciences, Iran. *; 4 *Department of Pathology, Kurdistan University of Medical Science, Sanandaj, Iran. *; 5 *Department of Health Research Methods, Evidence and Impact, McMaster University, Hamilton, ON, Canada. National Pain Centre, Department of Anesthesia, McMaster University, Hamilton, ON, Canada.*

**Keywords:** Nasopharyngeal Neoplasms, Cysts, Covid-19, Case Reports

## Abstract

**Introduction:**

The majority of nasopharyngeal cysts are asymptomatic and rare. Nevertheless, these lesions are rarely discovered during regular endoscopies and imaging tests. An upper nasopharyngeal Thornwaldt cyst is a benign, mucosal congenital cyst. Even less frequently, they can cause unexplained sinonasal symptoms such as rhinorrhea, vision problems, and nasal blockage.

**Case Report:**

Here, we report a case with new-onset hearing loss after the coronavirus infection, in which his imaging investigation showed a nasopharyngeal mass.

**Conclusion:**

After covid-19 infection we should consider branchial Cyst and Thornwaldt Cyst in a high-risk patients. On the other hand, the progressive hearing loss after covid-19 can occur due to activation of this kind of cysts.

## Introduction

Tinnitus, balance abnormalities, smell and taste disorders, as well as other otoneurological symptoms, are all signs that SARS-CoV-2 may be involved. However, research on Hearing Loss in COVID-19 patients is still in its infancy. It may be possible to address therapeutic options to enhance clinical recovery and prevent side effects by looking into the function of SARS-CoV-2 as an aetiological cause of Sudden Sensorineural Hearing Loss (SSNHL)(1). Viral infection is one explanation of SSNHL, according to research. The pathophysiology of this condition is not well established or clearly shown by studies(2).

Mass lesion of the nasopharynx is due to so many reasons. One of them is a branchial cleft cyst which is a rare disease. A branchial cyst is a congenital neck abnormality caused by the failure of the branchial apparatus to close entirely during embryogenesis (3). These cysts typically develop anterior to the sternocleidomastoid (SCM) muscle. Even though it is a congenital abnormality, most cases are not identified until the patient is in their 20s or 40s(4). They are divided into four types based on fetus cleft. First branchial cleft abnormalities are rare. The aberrant trapping of epithelium during the fusion of the six hillocks of His that create the external pinna causes skin tags, preauricular cysts, and sinus tracts around the ear. They're entirely ectodermal.95% of branchial abnormalities are second cleft lesions. Ectodermal and mesodermal cysts, sinuses, fistulas, and other combinations contain squamous epithelium, adnexal structures, or cartilage. Mucoid discharge or recurring infection typically alerts the patient. The turbid fluid makes the cyst non-transilluminate. Cysts in the second decade and sinuses in the first. Third branchial cysts are infrequent. The thyroid gland may cause the Cyst on the left side of the neck. Fourth branchial cleft anomalies are infrequent. A low-neck cyst or fistula may result (5-7). 

Thornwaldt Cyst is another midline nasopharyngeal Cyst that is rare too. Thornwald cysts arise when the notochord and the nasopharyngeal endoderm have a fetal communication, resulting in a benign congenital cyst in the pharyngeal bursa. They develop when the pharyngeal bursa becomes inflamed, swollen, or infected. The nasopharyngeal mucosa covers them and is superficial to the superior constrictor muscle (8). The vast majority of cases exhibit no symptoms at all. Nasal blockage, a feeling of a foreign mass in the nose, hearing loss, and intermittent bad breath are rare symptoms that patients may report (9).

We report a 40 years old male complaining about progressive both-side hearing loss after covid-19 infection. He had a nasopharyngeal branchial cyst on the right side and thornwaldt Cyst in the midline, which has been reported in a patient for the first time.

## Case presentations

**Fig 1:**
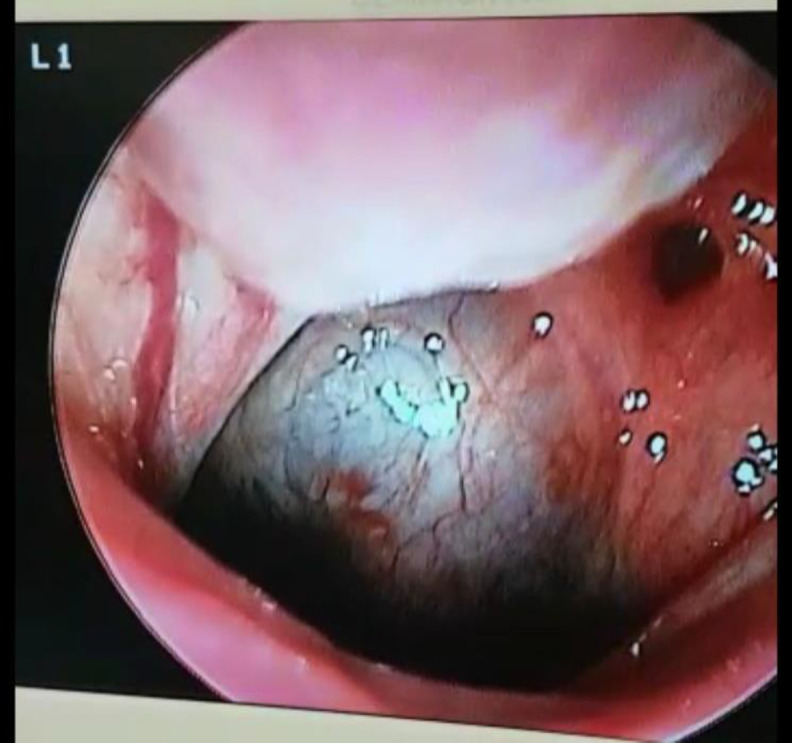
endoscopic view of the nasopharyngeal mass

A 40 years old male without previous medical history came to our clinic complaining about progressive both-side hearing loss since one month ago after a coronavirus infection. He was a health worker, and a nasopharyngeal PCR test had confirmed his coronavirus infection. Other physical exams were normal. Audio tympanometry showed bilateral conductive hearing loss (SRT: 40, GAP: 25 for the right side and SRT: 25, GAP:15 for the left side). Nasal endoscopy showed a well-defined smooth surfaced cystic mass in his posterior lateral and midline nasopharynx (Figure 1). 

**Fig 2:**
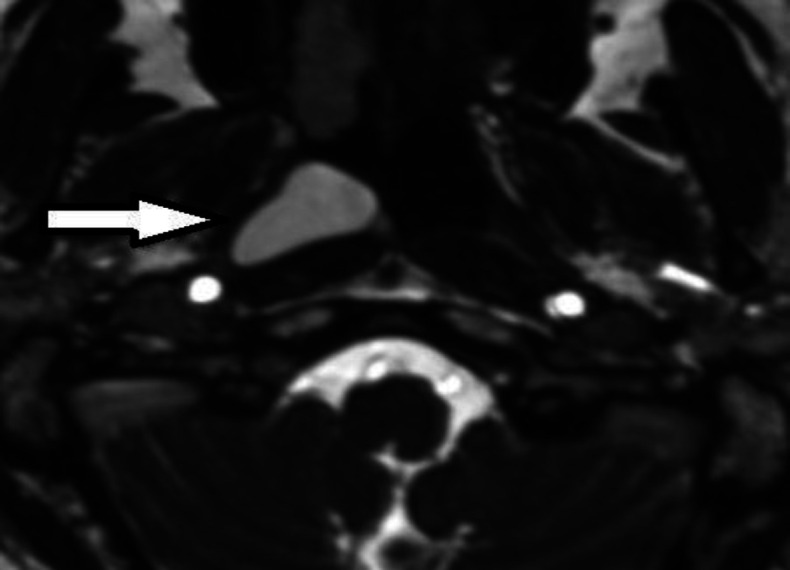
T1 (Right)and T2 (Left) high signal intensity lesion at a right posterolateral wall of nasopharynx suggestive of complicated or high protein containing brachial cleft cyst type 3 is seen

MRI with gadolinium showed a high signal intensity lesion at the nasopharynx's right posterolateral wall, suggestive of complicated or high protein-containing brachial cleft cyst type 2 based on its place (Figure 2). 

**Fig 3:**
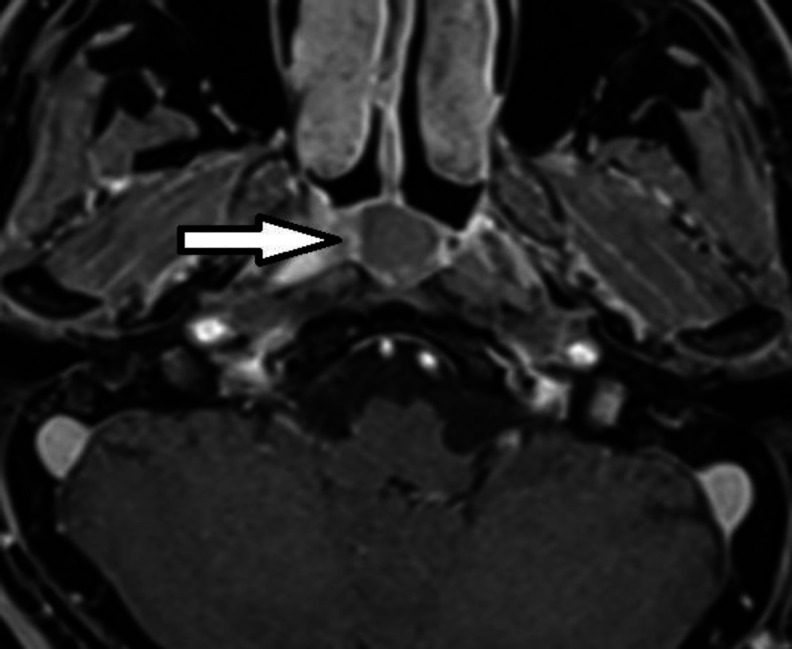
T1 high signal intensity cyst(right) with ring enhancement(left) at the central part of posterior wall of nasopharynx suggestive of complex or high protein containing thornwaldt Cyst is seen

It also showed a high signal intensity cyst with ring enhancement at the central part of the nasopharynx's posterior wall, suggestive of a complicated or high protein-containing thornwaldt cyst (Figure 3). 

**Fig 4:**
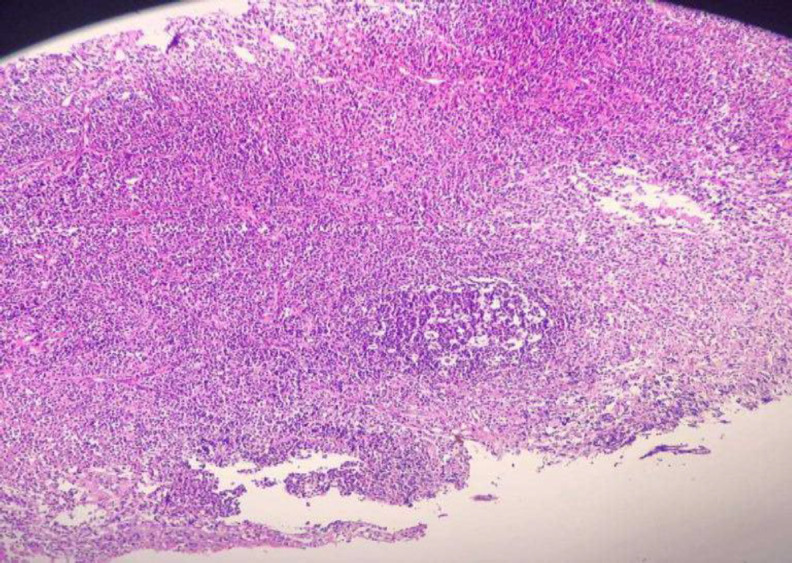
right (lymphoid follicle with the germinal center). Left (squamous epithelial lining with underlying lymphocytic infiltration)

 He underwent surgery with the diagnosis of a branchial cyst, and marsupialization was performed under general anesthesia; Cyst was full of Brawny discharge. Biopsy was taken, and pathology showed fragments of cyst-like structure lined by stratified squamous and respiratory ciliated epithelium with underlying chronic inflammation lymphoid aggregation and fibrosis (Figure 4). 

 It was compatible with the branchial cleft cyst. After one year of follow-up, there is no recurrence, and the patient is asymptomatic.

## Discussion

The nasopharynx should unquestionably be assessed before administering any medicinal therapy to patients who complain of nasal blockage and have no neurological symptoms. This case is the first reported case of a tornwaldt Cyst and nasopharyngeal branchial cleft cyst simultaneously. They both are rare diseases, and their management is surgical in both. When the bursa's entry to the nasopharynx is blocked by an infection, an adenoidectomy, or radiation therapy, a Thornwaldt cyst forms. On the other hand, Depending on their infectious status, bronchogenic cysts may present with symptoms. In this patient, we suggest that the covid-19 infection had a role in Thornwaldt cyst formation and bronchial cyst presentation due to the lack of symptoms before infection.

Histopathologically, branchial cysts are lined by various types of epithelium, mostly stratified squamous and ciliated columnar epithelium (10). We have a second branchial cleft anomaly based on our pathology (Figure 2). The second branchial cleft anomaly is the most common type, representing about 40-95% of this anomaly. It can be seen from the neck anterior to the sternocleidomastoid muscle to the tonsillar fossa. In 1955 Proctor classified the second branchial cleft cyst into four types. Type one is located superficially on the anterior border of SCM and beneath the cervical fascia. The most common type is under the investing layer of deep cervical fascia. Type three passes between the carotid arteries and can extend to the pharyngeal wall. Type four lies next to the pharyngeal wall, medial to the great vessels (11). The nasopharyngeal location of the branchial Cyst is unusual, and a few dozens of case report has mentioned it. According to Segal et al. l, the average age of patients with NBC was 36 years, mostly reported from Asia (10). Tornwaldt cyst is primarily tiny and remains undiagnosed, but it can grow, and symptoms like nasal obstruction, postnasal drip, headache, neck stiffness, and Eustachian tube dysfunction can occur (12). Clinically, there are two categories for thornwaldt cysts. The first form is the more prevalent cystic variety, which cannot naturally drain into the nasopharynx. The other type is crusting, which discharges into the nasopharynx regularly and randomly (13,14). The case in our study was found to be typical of the cystic form. Nasal endoscopy illustrates a well-defended mass in the midline of the nasopharynx. MRI with GAD is diagnostic. If it is asymptomatic, no intervention is required, but surgical intervention is curative in symptomatic cases (15). The goal of treatment is to reduce mass effect symptoms and take pathology to rule out malignancy (16).

Clinical presentation varies based on the size and extension of the Cysts. These cysts are primarily small and asymptomatic. in symptomatic cases, Ear fullness, serous otitis media, and conductive hearing loss are common (17). Nasal obstruction and rhinorrhea are also reported (10). In our case, the chief complaint was ear fullness and hearing loss due to the mass extension.

Unfortunately, the occurrence has not been reported till now. There are different techniques to treat, including aspiration, excision, or marsupialization. These recent years there has been a tendency to use endoscopic/robotic approaches in rich countries (16,18). However, this kind of surgery is not used in Iran yet, so the patient was treated with a traditional endoscopic approach.

## Conclusions

Before administering any medical treatments to individuals who simply have the complaint of new onset hearing loss and have no neurological signs or active infection, the nasopharynx could be thoroughly examined. After covid-19 infection we should consider branchial Cyst and Thornwaldt Cyst in a patient with risks. On the other hand, the progressive hearing loss after covid-19 can occur due to activation of this kind of cysts.
